# Hibernation in the pygmy slow loris (*Nycticebus pygmaeus*): multiday torpor in primates is not restricted to Madagascar

**DOI:** 10.1038/srep17392

**Published:** 2015-12-03

**Authors:** Thomas Ruf, Ulrike Streicher, Gabrielle L. Stalder, Tilo Nadler, Chris Walzer

**Affiliations:** 1 Department of Integrative Biology and Evolution, Research Institute of Wildlife Ecology, University of Veterinary Medicine, Vienna, Austria; 2 Endangered Primate Rescue Center, Cúc Phương, Ninh Bình, Vietnam; 3 1185 East 39th Place, Eugene, Oregon, USA

## Abstract

Hibernation and short daily torpor are states of energy conservation with reduced metabolism and body temperature. Both hibernation, also called multiday torpor, and daily torpor are common among mammals and occur in at least 11 orders. Within the primates, there is a peculiar situation, because to date torpor has been almost exclusively reported for Malagasy lemurs. The single exception is the African lesser bushbaby, which is capable of daily torpor, but uses it only under extremely adverse conditions. For true hibernation, the geographical restriction was absolute. No primate outside of Madagascar was previously known to hibernate. Since hibernation is commonly viewed as an ancient, plesiomorphic trait, theoretically this could mean that hibernation as an overwintering strategy was lost in all other primates in mainland Africa, Asia, and the Americas. However, we hypothesized that a good candidate species for the use of hibernation, outside of Madagascar should be the pygmy slow loris (*Nycticebus pygmaeus*), a small primate inhabiting tropical forests. Here, we show that pygmy slow lorises exposed to natural climatic conditions in northern Vietnam during winter indeed undergo torpor lasting up to 63 h, that is, hibernation. Thus, hibernation has been retained in at least one primate outside of Madagascar.

Over the past decades, it has become clear that heterothermia and hypometabolism in endotherms, i.e., torpor and hibernation, are not restricted to temperate or arctic conditions. Lemurs, rodents, bats, marsupials, and monotremes use torpor in subtropical or tropical habitats, which can be quite seasonal and energy demanding^1^,^2^,^3^,^4^,^5^. It seemed unlikely therefore, that the unusual situation among primates, in which true hibernation seemed to be restricted to Malagasy lemurs, should actually reflect the complete absence of primate hibernators in other parts of the world. We hypothesized that any non-Malagasy primate using torpor should be found in a habitat with clear seasonal changes in temperature and/or precipitation as well as seasonally changing food availability. Further, we predicted that such a primate would be relatively small, because hibernation in bears and other large carnivores is the rare exception, whereas the vast majority of hibernators have body masses <3.5 kg (median 70 g^6^). Finally, we expected that this putative primate hibernator would probably belong to the suborder Strepsirrhini, which also includes all Malagasy lemurs that exhibit torpor^5^ as well as the galagos, such as the African lesser bushbaby (*Galago moholi*), which uses daily torpor as an emergency response^7^.

All three of these criteria apply to pygmy slow lorises, which are small strepsirrhine primates inhabiting forests in Vietnam, Cambodia, Laos and China with mostly quite seasonal environments. In northern Vietnam, for example, the climate is profoundly seasonal with wet warm summers and cool dry winters during which ambient temperature (T_a_) reaches 5 °C^8^. Given their relatively small body size (~400 g) and associated high rates of heat loss, low winter temperatures can be expected to create high energetic costs for thermoregulation in pygmy slow lorises. However, during winter this omnivorous species also faces a shortage of fresh vegetation and insects^9^. During this time of the year, normally nocturnally active pygmy slow lorises are known to remain entirely inactive for prolonged periods of time, which would be compatible with their use of torpor^10^. There are anecdotal reports of animals that appeared to be torpid, that is, stiff, unresponsive, and cool to the touch^8^,^10^, but this was not corroborated by any measurements of body temperature (T_b_) or metabolism. Moreover, this species is known to exhibit an apparently endogenously controlled body mass gain of up to 50% towards winter, as is typically seen in fattening hibernators^10^. Thus, a variety of factors pointed to a possible occurrence of heterothermia in pygmy slow lorises, and its adaptive potential. To see if *N. pygmaeus* in fact displays any form of torpor we continuously measured core body temperatures using intraperitoneally implanted data loggers over fall, winter, and subsequent spring. The animals were kept in large outdoor enclosures, which allowed for both recapture and veterinary surveillance, but were exposed to natural climatic conditions in northern Vietnam.

## Results

**Figure 1:**
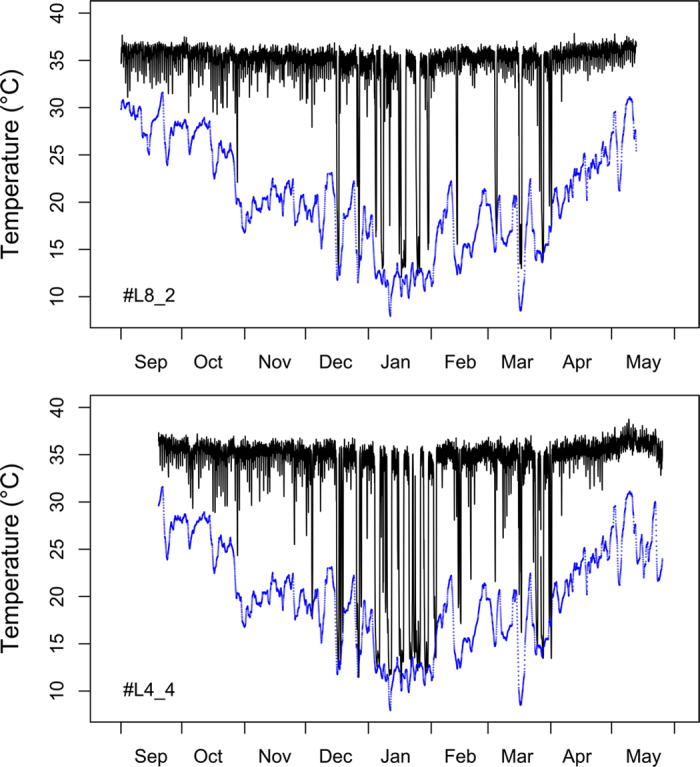
Core T_b_ (black lines) recorded over ~9 months at 6 min intervals in two pygmy slow lorises living in outdoor enclosures is northern Vietnam. Drops of T_b_ below 30 °C during winter represent episodes of torpor. Blue dots show air temperature, smoothed by a 12-h moving average to improve clarity.

**Figure 2:**
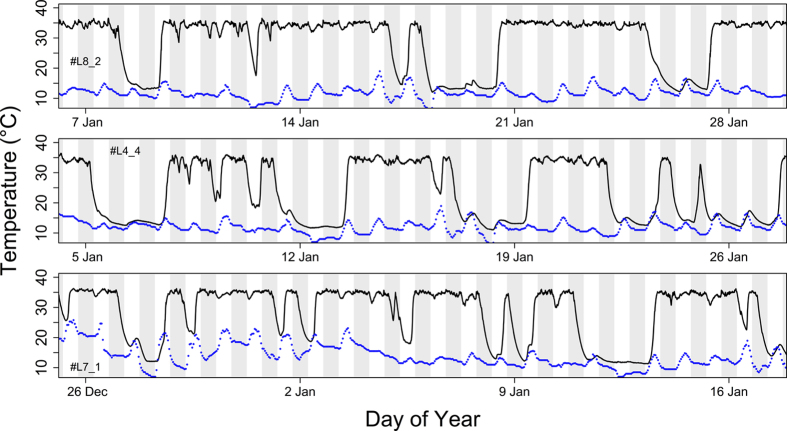
Core T_b_ (black lines) in three pygmy slow lorises in midwinter, recorded over three-week periods. All animals hibernated, that is, exhibited bouts of multiday torpor (up to 63 h) that were interspersed with periods of euthermia and short torpor episodes. Blue dots show air temperature. White and grey areas show day and night, respectively.

All animals monitored over winter showed bouts of torpor, with the first bout occurring during a cold spell in late October and the last one in early April (Fig. 1). Torpor was most frequent in midwinter (mid December – mid February; Fig. 1). During this period the animals exhibited multiday torpor, i.e., hibernation, interspersed with several days of euthermia and/or short torpor episodes (Fig. 2). On euthermic days between torpor bouts, all animals fed on the food provided in the enclosures.

Mean overall torpor bout duration was 15.0 ± 1.8 h (n = 91). Bouts of multiday torpor lasted on average 43.0 ± 3.0 h (n = 20; range: 25.9–62.6 h). The mean minimum T_b_ reached during torpor was 22.6 ± 0.6 °C (n = 91; range: 11.0–29.9 °C). Minimum T_b_s were similar in all animals, i.e., 12.0 °C, 11.0 °C, 11.0 °C and occurred at T_a_s of 6.5 °C, 6.0 °C, and 8.0 °C, respectively. Times of torpor entrances were not uniformly distributed (Rayleigh’s test P < 0.001) but predominantly occurred in the early morning (mean: 05:19 ± 0.21 h). Times of arousals also significantly clustered around a mean of 14:06 ± 0.19 h (Rayleigh’s test P < 0.001).

**Figure 3:**
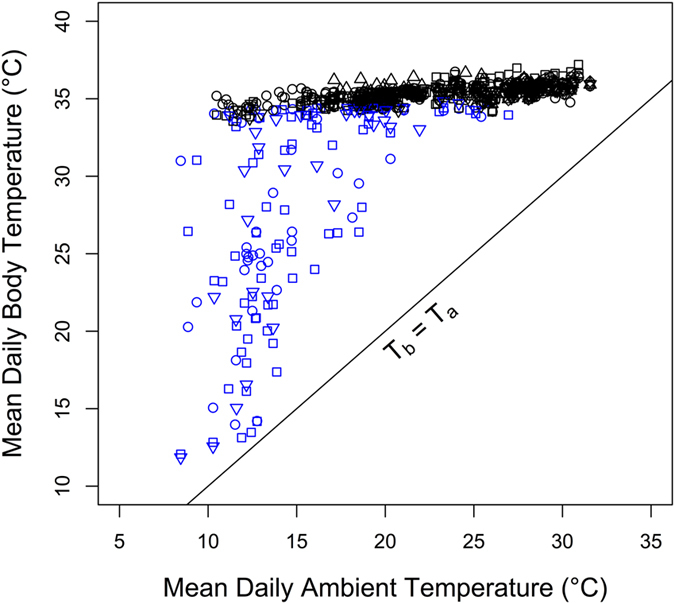
Mean daily T_b_ as a function of mean daily T_a_ in five pygmy slow lorises (n = 769 days). Mean T_b_ decreased with mean T_a_ both on days with torpor (blue symbols; minimum T_b_ < 30 °C) and on euthermic days (black symbols). Different symbols denote different animals. Note that at mean daily T_a_s below ~13 °C, T_b_−T_a_ gradients increased, which may indicate active thermogenesis to maintain minimum T_b_.

Lorises were more prone to enter torpor on cold days. The probability to undergo torpor significantly increased as mean T_a_ (computed over 24 h prior to noon of the day of torpor entry) decreased (binomial model; z = −11.1, P < 0.001). Further, mean daily T_b_ was positively associated with T_a_ both on days with torpor (daily minimum T_b_ < 30 °C) and on euthermic days (Fig. 3.). On days with torpor mean daily T_b_, as an integrative measure of both depth and duration of torpor, significantly decreased with mean daily T_a_ (T_b_ = 13.1 + 0.11*T_a_; R^2^ = 0.36; P < 0.001). During euthermic days, mean T_b_ obtained from all 5 animals investigated also was a linear function of mean T_a_ (T_b_ = 33.7 + 0.07*T_a_; R^2^ = 0.44; P < 0.001). Interestingly, this was also the case for maximum daily T_b_ (T_b_ = 35.29 + 0.056*T_a_; R^2^ = 0.36; P < 0.001; data not shown on graph).

## Discussion

We were able to obtain T_b_ records from a total of five animals, including three animals recorded during winter. Despite this limited number of individuals, our results leave no doubt that *N. pygmaeus* has the capability to hibernate, as all animals (of both sexes) monitored during winter did undergo bouts of multiday torpor (Fig. 2). Hibernation is defined by the occurrence of torpor bouts that last longer than 24 hours (e.g.^11^,^12^), which clearly is the case in pygmy slow lorises. This definition of hibernation is justified because, whilst several hibernators may also exhibit short torpor bouts lasting only several hours, the opposite is not the case: Many mammals may undergo daily torpor but are incapable to exhibit multiday torpor, which requires releasing metabolism and T_b_ from the strict control by the circadian pacemaker (review in^6^). Daily torpor seems to be a mere amplification of daily rhythms in metabolism and T_b_, whereas hibernation requires switching to an alternative clock mechanism that allows staying torpid over multiple days^6^,^13^,^14^. Therefore, there is a growing consensus that daily torpor represents the more ancient trait, whereas prolonged hibernation is viewed as an advanced, secondary adaptation^6^,^14^,^15^,^16^. Apparently, this evolutionary step has occurred in *N. pygmaeus* and it would not be surprising if future studies reveal that hibernation occurs in further primates outside Madagascar, especially within the Lorisidae.

We have several reasons to think that spontaneous torpor and hibernation during episodes of low T_a_, as observed here, are natural responses in *N. pygmaeus*, and were not caused by our keeping the animals in outdoor enclosures. The animals in our study were well acclimated to living in enclosures. Also, even though torpor may be induced by shortage of food, the experimental animals were continually offered and in fact consumed a broad spectrum of food items throughout our study. Thus there is no indication that lorises were food restricted or nutritionally stressed. Further, there is accumulating evidence that species that can be very reluctant to exhibit torpor in captivity regularly do so in the wild^6^. Therefore, if our housing conditions had any effect at all, we would expect that they might have lowered the use of torpor, compared with free-ranging animals. Finally, there have been anecdotal observations pointing to torpor in free-living pygmy slow lorises before^8^.

The highly variable pattern of multiday torpor of lorises, interspersed with several days of euthermia and short torpor bouts (Fig. 2), seems to be quite typical for tropical species that show torpor at relatively high T_a_^4^,^17^. However, when provided with food during winter even north-temperate hibernators, such as the garden dormouse, can show very similar patterns and may remain active for several days between torpor episodes^18^. The fact that the use of torpor in pygmy slow lorises was apparently limited to cooler periods suggests that heterothermia was employed opportunistically and restricted to environmental conditions that would have required large energy expenditure for thermoregulation. Also, none of the five animals showed torpor outside the winter season and hibernation was restricted to the coldest months (Figs 1, 2). These findings support the view that, while torpor results in enormous energy savings, it also involves risks and trade-offs^19^,^20^,^21^,^22^. In pygmy slow lorises, a particularly important risk of torpor may be increased susceptibility to predation. Although pygmy slow lorises move slowly and are unable to leap even when euthermic^9^, they will be entirely unable to escape predators in the torpid state. However, this handicap may be at least partially compensated by the fact that the animals are relatively well camouflaged^9^ and are motionless and hardly emit any odour while torpid, which makes them difficult to detect for predators^23^.

The benefits of torpor in terms of energy savings, on the other hand, are evident, even if metabolic rate was not measured in this study. Especially when T_b_ is lowered to values very close to T_a_, as in pygmy slow lorises (Figs 2, 3), hibernation results in a reduction of energy expenditure to ~5% of the animals’ basal metabolic rate^6^. In pygmy slow lorises a drastic reduction of energy expenditure is apparently an adaptation to the seasonal change in food availability, in particular the low abundance of insects in winter. As indicated by the large seasonal change of body mass in this species^10^, which peaks in November/December (578 g ± 58 SD in males, 543 g ± 111 SD in females) and is lowest in March/April (367 g ± 26 SD in males, 360 g ± 25 SD in females)^10^, it seems that pygmy slow lorises largely rely on body energy stores during winter similar to other tropical hibernators^24^. However, as the animals in our study were offered food throughout the year, torpor and hibernation are most likely not directly triggered by changes in food availability. In other mammals, the seasonal occurrence of hibernation and daily torpor is controlled by an endogenous circannual clock or photoperiodic time measurement^13^,^25^,^26^, which may be also the case in *N. pygmaeus*.

One interesting aspect of hibernation in pygmy slow lorises, and other tropical hibernators^4^, is that they remain exposed to large daily fluctuations in T_a_, whereas many ‘classical’ hibernators experience almost constant T_a_ in their underground burrows (e.g.,^27^). This factor, together with continued nocturnal foraging activity, has apparently led to a synchronization of torpor with the light-dark cycle. Pygmy slow lorises predominantly entered torpor during late night/early morning, when lowering the large T_b_-T_a_ gradient is energetically most effective. Even more interestingly, return to euthermia (T_b_ > 30 °C) occurred typically in the early afternoon, that is, well prior to the onset of nocturnal activity^9^. We attribute this timing to the energetic advantage of synchronizing rewarming with the daily rise in T_a_, which is quite obvious from the temperature traces in Fig. 2. It is well known that several other heterotherms make use of passive warming during rises in T_a_ and even use sun-basking to minimise the energetic costs of returning to euthermia^28^,^29^. During the cold season, pygmy slow lorises indeed move to sunny spots during their daytime resting phase, whereas during hot periods they only change sleeping sites when disturbed (U.S. unpublished observations).

In addition to the use of torpor and hibernation, pygmy slow lorises also reduced energy requirements by significantly lowering both mean and maximum daily T_b_, even in the euthermic state, as T_a_ declined (Fig. 3). Within a smaller range of T_a_s this linear decrease of T_b_ in *N. pygmaeus* has been observed before^30^. The degree of down-regulation of T_b_ (~1.5 °C over a 20 °C T_a_ range) even during the daily active phase is unusual for primates^31^,^32^,^33^ but interestingly has also been observed in the closely related slow loris, *Nyctycebus coucang*^34^. Whether the pronounced seasonal change of pelage in lorises^9^ also plays a role for thermoregulation is currently unknown. However, it seems that pygmy slow lorises employ an entire arsenal of mechanisms to minimise energy expenditure during winter, ranging from reduced activity^9^, over lowered euthermic T_b_, to torpor and hibernation. It would be interesting to see if these physiological responses are even more pronounced in entirely free-living animals that have to rely on natural food resources only.

In summary, this study shows that hibernation in primates is not restricted to species inhabiting Madagascar. Thus, there are no specific climatic conditions or evolutionary histories that have limited the occurrence of primate hibernation to this island. This finding opens the door for the search for hibernation in further primates living in seasonal habitats.

## Material and Methods

### Animals and study site

The study was carried out between August 2010 and May 2011 at the Endangered Primate Rescue Center (EPRC), Cúc Phương, Ninh Bình, Vietnam (20°14′N , 105°44′E) using 6 adult pygmy slow loris (4 non-pregnant females, 2 males; body mass 240–420 g). The animals had been confiscated by Vietnamese authorities from illegal wildlife traders, and were kept at EPRC in outdoor enclosures for at least 3 months prior to the experiments. All experiments were carried out in accordance with the guidelines on “Use if Animals in Research and Education” of the World Organization on Animal Health (OIE), of which Vietnam is a member. All experimental protocols were approved by the Vietnamese Ministry of Agriculture and Rural Development, Decision 59/QD-BNN-KL (dated 09.09.2009). All experimental procedures were also reported to the Ethics Committee of the University of Veterinary Medicine, Vienna, Austria. Animals were housed individually in outdoor enclosures (size 3 m*1.5 m*2 m) with open soil, natural vegetation of a tropical evergreen forest and a furnishing of bamboo and branches. Sleeping boxes were provided as shelters. The animals were fed the same mixture of boiled eggs, fruits and vegetables (bananas, pineapples, dragonfruit, papaya, apples, pears, oranges, grapes and carrots) throughout the year. In addition, stick insects, grasshoppers and crickets were fed daily (except for periods of rainy weather of up to three days) additionally between May and October, when these insects were abundant, but not during winter, when arthropods are virtually absent in this habitat^8^. In addition, the natural vegetation and natural soil in the enclosures allowed for a certain degree of independent capture of insects. The animals had permanent access to water. Air temperature at the site was measured at hourly intervals using a data logger (EL-USB2, Lascar Electronics, Salisbury, UK; accuracy ± 0.5 °C) placed inside one of the enclosures in the shade.

### Body temperature measurements

Core T_b_ was measured at 6 min intervals using intraperitoneally implanted, custom-constructed programmable data loggers based on digitally readable temperature sensors (MCP 9800, Microchip Technology, Chandler, AZ, USA; resolution: 0.0625 °C storage capacity: 104 832 values; weight 6.5 g). Loggers were individually calibrated in a water bath prior to implantation and checked for accuracy after explantation. The maximum deviation between measured and actual temperature after explantation was <0.2 °C. Prior to implantation, loggers were coated with a paraffin/beeswax mixture and subsequently EtO-gas-sterilized (Anprolene AN74i, Andersen Products Inc., Haw River, NC, USA). However, apparently due to faults in the coating several of the loggers failed after implantation (but retained the stored data). Thus, we obtained records for the full study period (September-May) for 2 out of 6 implanted loggers only (one male, one female), as well as data for September to mid January for one female. For two more females, we retrieved records from autumn (September-October) over 45 and 80 days, respectively. One logger failed after a few days only, and these data were entirely omitted. All together, T_b_ was measured during 769 days in 5 animals (255, 250, 139, 80, and 45 days per animal).

### Surgery

Surgical anaesthesia was induced by intramuscular injection of 5 or 10 mg kg^−1^ ketamine (Ketamidor 10%; Richter Pharma, Wels, Austria) and 50 μg kg^−1^ medetomidine (Dormitor; Orion Parma, Espoo, Finland) and maintained with 0.2–1.5% isoflurane (IsoFlo; Abbott Laboratories, Abbott Park, Il, USA) in an oxygen stream. The animals were placed in dorsal recumbency, the operation field was prepared according to standard surgical procedures and covered with sterile surgical drapes. The abdominal cavity was opened through a ~1.5 cm incision in the central midline. The logger was inserted free-floating. Postimplantation, peritoneum, abdominal muscles were sutured using synthetic absorbable surgical suture material USP 3/0 (Surgicryl PGA; SMIAG, Hunningen, Belgium) using a single-button suture technique. The skin was sutured separately using synthetic absorbable surgical suture material USP 3/0 (Surgicryl PGA; SMI AG) with an intracutaneous suture technique. During the entire procedure, vital parameters [respiration rate, peripheral haemoglobin oxygen saturation as measured by pulse oximetry (SpO2), heartrate] were monitored. Postsurgical analgesia (5 mg kg^−1^ Meloxicam (Kwizda Pharma, Vienna, Austria) subcutaneously) was provided.

### Data analysis

Torpor was defined as T_b_ decreasing below 30 °C for at least 1 h. This slightly conservative definition was used because T_b_ was highly variable even at night in the absence of clear torpor episodes (Figs 1,2), and in order to identify events that were most likely energetically relevant. In the three animals measured in winter we observed 91 torpor episodes. As there were no notable differences in torpor characteristics among animals, summary statistics were computed from pooled data. Data are given as means ± SEM. To see if times of torpor entrance and arousal were uniformly distributed we used Rayleigh’s test as provided by the R-package “circular”^35^. For the relationships between mean daily T_b_ as well as maximum daily T_b_ and T_a_ on euthermic days we fitted linear mixed effects models, using animal-ID as a random factor to adjust for repeated measurements (R-package nlme)^36^. To test if T_a_ had an effect on the probability of animals to enter torpor we computed a variable torpor (yes/no) for each day (n). We then computed mean T_a_ for the period 12:00 h on day n−1 to 12:00 h on day n, and tested the effect of this mean T_a_ on the probability of torpor entry on day n using a mixed effects binomial model (R package lme4)^37^, again entering animal ID as the random effect. There was no indication for overdispersion in this model (dispersion parameter 1.08).

## Additional Information

**How to cite this article**: Ruf, T. *et al*. Hibernation in the pygmy slow loris (*Nycticebus pygmaeus*): multiday torpor in primates is not restricted to Madagascar. *Sci. Rep*. **5**, 17392; doi: 10.1038/srep17392 (2015).
